# Genome-wide identification of biotin carboxyl carrier subunits of acetyl-CoA carboxylase in *Brassica* and their role in stress tolerance in oilseed *Brassica napus*

**DOI:** 10.1186/s12864-022-08920-y

**Published:** 2022-10-17

**Authors:** Swati Megha, Zhengping Wang, Nat N. V. Kav, Habibur Rahman

**Affiliations:** Department of Agricultural Food and Nutritional Science, University of Alberta, Edmonton, AB T6G 2P5 Canada

**Keywords:** BCCP, Brassica, Phylogeny, Stresses, *Plasmodiophora brassicae*, *Sclerotinia sclerotiorum*

## Abstract

**Background:**

Biotin carboxyl carrier protein (BCCP) is a subunit of Acetyl CoA-carboxylase (ACCase) which catalyzes the conversion of acetyl-CoA to malonyl-CoA in a committed step during the de novo biosynthesis of fatty acids. Lipids, lipid metabolites, lipid-metabolizing and -modifying enzymes are known to play a role in biotic and abiotic stress tolerance in plants. In this regard, an understanding of the *Brassica napus BCCP* genes will aid in the improvement of biotic and abiotic stress tolerance in canola.

**Results:**

In this study, we identified 43 *BCCP* genes in five *Brassica* species based on published genome data. Among them, *Brassica rapa*, *Brassica oleracea*, *Brassica nigra*, *Brassica napus* and *Brassica juncea* had six, seven, seven, 10 and 13 *BCCP* homologs, respectively. Phylogenetic analysis categorized them into five classes, each with unique conserved domains. The promoter regions of all *BCCP* genes contained stress-related cis-acting elements as determined by cis-element analysis. We identified four and three duplicated gene pairs (segmental) in *B. napus* and *B. juncea* respectively, indicating the role of segmental duplication in the expansion of this gene family. The Ka/Ks ratios of orthologous gene pairs between *Arabidopsis thaliana* and five *Brassica* species were mostly less than 1.0, implying that purifying selection, i.e., selective removal of deleterious alleles, played a role during the evolution of *Brassica* genomes. Analysis of 10 *BnaBCCP* genes using qRT-PCR showed a different pattern of expression because of exposure of the plants to biotic stresses, such as clubroot and sclerotinia diseases, and abiotic stresses such as drought, low temperature and salinity stresses.

**Conclusions:**

The identification and functional analysis of the *Brassica* BCCPs demonstrated that some of these genes might play important roles in biotic and abiotic stress responses. Results from this study could lay the foundation for a better understanding of these genes for the improvement of *Brassica* crops for stress tolerance.

**Supplementary Information:**

The online version contains supplementary material available at 10.1186/s12864-022-08920-y.

## Background

In plants, the biosynthesis of triacylglycerols is critical for the accumulation of seed oil. Fatty acid biosynthesis in plants occurs within plastids and is catalyzed by two enzymes, acetyl-CoA carboxylase (ACCase) (E.C. 6.4.1.2) and fatty acid synthase. ACCase catalyzes the first committed step in the de novo biosynthesis of fatty acid, the carboxylation of acetyl-CoA to malonyl-CoA through ATP-dependent carboxylation of acetyl-CoA [1]. In nature, two physically distinct types of ACCase, viz. heteteromeric and homomeric, exists. In the case of heteromeric ACCase, the following four components, viz. biotin carboxylase (BC; EC 6.3.4.14), biotin carboxyl carrier protein (BCCP), and α- and β-subunits of carboxyltransferases (CTs), are required for their activity, and these components are expressed as individual subunits that form a multienzyme complex and are usually present in the plastids of algae, bryophytes, gymnosperms, dicotyledonous plants as well as in archaea [1–4]. In case of the homomeric ACCase, all four enzymatic components are translated as a single polypeptide, and this form is predominantly involved in the de novo biosynthesis of fatty acids in the cytosol of animals and fungi, and in the cytosol of dicots, as well as the cytosol and plastids of graminaceous monocots [3]. In plants, plastid ACCase (ACCase1) is involved in the biosynthesis of long-chain fatty acids, while cytosolic ACCase (ACCaase2) is important for secondary metabolism, including the synthesis of very long-chain fatty acids (VLCFA), flavonoids, cuticular waxes, and other compounds, and for proper embryonic development [5–7]. This compartmentation of ACCases in plastids and cytosol may be necessary for the regulation of the amount of malonyl-CoA and other reactions [7].

The reaction catalyzed by heteromeric ACCase can be divided into two different half-reactions: the ATP-dependent carboxylation of biotin using bicarbonate followed by the transfer of the carboxyl group to acetyl-CoA [8]. Both these reactions are facilitated by the low molecular weight protein cofactor, BCCP, which contains a biotin prosthetic group covalently linked to a lysine residue within a conserved biotinylation sequence motif (CIIEAMKLMNEIE or CIVEAMKLMNEIE) in the C-terminal region [9, 10].

$$\mathrm{BCCP}-\mathrm{biotin}+\mathrm{HCO}^{-3}+\mathrm{Mg}^{2+}-\mathrm{ATP}\rightarrow\mathrm{BCCP}-\mathrm{Biotin}-\mathrm{CO}^{-2}+\mathrm{Mg}^{2+}-\mathrm{ADP}+\mathrm{Pi}(\mathrm{catalyzed\; by\; BC\; subunit})$$

$$\mathrm{BCCP}-\mathrm{biotin}-\mathrm{CO}^{-2}+\mathrm{acetyl}-\mathrm{CoA}\rightarrow\mathrm{BCCP}-\mathrm{biotin}+\mathrm{malonyl}-\mathrm{CoA}(\mathrm{catalyzed\; by\; CT\; subunit})$$

In *Arabidopsis thaliana*, two paralogous copies of *BCCP* are present, *AtBCCP1* and *AtBCCP2* exhibiting 42% amino acid sequence identity. *AtBCCP1* is reported to be constitutively expressed in all tissues, while *AtBCCP2* transcript is predominantly present in flowers and siliques [11, 12]. The *Brassica napus* genome has been reported to contain at least six *BCCP* copies forming two distinct classes based upon amino acid and nucleotide sequence comparisons [13]. *AtBCCP1* has been reported to be most similar to class one oilseed rape BCCP subunits, while *AtBCCP2* is homologous to class two *BCCP*s of *B. napus* [12]. Additionally, genes encoding BCCP proteins have also been characterised and cloned from soybean [14], cotton [15], jatropha [16], *Vernicia fordii* [17], and *Aleurites moluccana* [9]. The role of BCCP proteins in the de novo biosynthesis of fatty acids was confirmed in *A. thaliana* by overexpression and reverse genetics [18]. Overexpression and antisense expression of the *BCCP2* in developing seeds resulted in reduced fatty acid content and higher linolenic acid levels at the expense of oleic and linoleic acids implying that overexpression of *BCCP2* may inhibit ACCase activity. Recently, 24 putative *BCCP* genes were identified in four cotton species and their expression pattern was analysed after exposure to cold and salinity stresses [10].

The Brassicaceae (mustard family) comprises approximately 340 genera and 3,350 species, including the economically important *Brassica* crops and the model organism *A. thaliana*. The genus *Brassica* of the tribe Brassiceae includes 39 species [19] possessing enormous morphological diversity and are used as a source of oil, vegetables, dietary fiber, and condiments. Among the *Brassica* species, *Brassica rapa* (2*n* = 20, AA), *B. nigra* (*2n* = 16, BB) and *B. oleracea* (*2n* = 18, CC) are three diploid progenitor species which led to formation of the allopolyploid species *B. napus* (*2n* = 38, AACC), *B. juncea* (*2n* = 36, AABB) and *B. carinata* (*2n* = 34, BBCC) [20]. The recent availability of completed genomic sequences of *B. rapa* [21], *B. oleracea* [22], *B. nigra* [23], *B. napus* [24], *B. juncea* [25], and *B. carinata* [26] provides us an excellent opportunity for genome-wide identification, evolution, and functional analysis of important gene families in these species.

Plants can adapt to a wide range of biotic (fungal, bacterial and insect pests) and abiotic (e.g., drought, salinity, temperature extremes) stresses by reprogramming their transcriptomes, proteomes, and metabolomes. Various -omics studies have revealed that complex regulatory networks are involved in mediating biotic and abiotic stress tolerance in plants [27–29], where lipids and lipid-metabolizing genes play an important role [30]. Plant lipids, lipid metabolites, and lipid-metabolizing and -modifying enzymes are also known to play important roles in disease resistance [31, 32] and abiotic stresses including drought and salinity [33–40] An understanding of the *Brassica BCCP* genes may, therefore, benefit researchers and breeders to improve the important oilseed crops for resistance to different biotic and abiotic stresses.

In this study, we performed the genome wide identification of *BCCP* genes in six *Brassica* species and carried out a comprehensive analysis of 43 genes based on gene structure, phylogeny, chromosomal distribution, conserved motifs, and cis acting elements in the promoter regions. We also examined the syntenic relationship of the *BCCP* genes between *Brassica* and *A. thaliana* as well as assessed the orthologous and paralogous relationships of the *Brassica BCCP* genes. Furthermore, we carried out an expression analysis of 10 *BCCP* genes in oilseed *B. napus* under different biotic stresses, such as infection with the pathogens *Plasmodiophora brassicae* (clubroot disease) and *Sclerotinia sclerotiorum* (stem rot disease), and abiotic stresses, such as cold, salinity and water-deficit stress mediated by polyethylene glycol (PEG) treatment, as well as after treatment with hormones (Salicylic acid, SA; Abscisic acid, ABA; and the cytokinin (CK), 6-Benzylaminopurine, BAP).

## Results

### Genome-wide identification of *BCCP* genes in *Brassica*

We identified all the putative *BCCP* genes in six *Brassica* species viz. *B. rapa*, *B. oleracea*, *B. nigra, B. napus*, *B. juncea* and *B. carinata* through BLASTP and BLASTN searches against their respective databases, using the query sequences *BCCP* genes from *A. thaliana*. In the case of *B. carinata*, BLASTP resulted in two hits; however, their chromosomal location could not be determined from the information in the database, hence we excluded the *B. carinata* sequences from further analysis. By using the InterProScan program and SMART database, the presence of the biotinylation domain (CIIEAMKLMNEIE or CIVEAMKLMNEIE or CYIEQLGGQFPIESDVTGEVVKI) was confirmed in the sequences, and based on this, a total of 43 *BCCP* genes were identified in the five *Brassica* species genomes (six in *B. rapa*, seven in *B. oleracea*, seven in *B. nigra*, 10 in *B. napus* and 13 in *B. juncea*; Supplementary File 2). The predicted *BCCP* genes, *BnaBCCP1* to *BnaBCCP10*, *BraBCCP1* to *BraBCCP*6, *BolCCP1* to *BolBCCP*7, *BjuBCCP1* to *BjuBCCP13*, and *BniBCCP1* to *BniBCCP*7 were numbered based on their location on the chromosomes and are presented in Table 1. Based on this, the number of *BCCP* genes that could be identified in the amphidiploid species *B. juncea* is exactly sum of the number of the genes that could be found in its two progenitor species *B. rapa* and *B. nigra*; however, the physical position of these genes in the amphidiploid and diploid genomes is linear in about 50% (6/13) of the cases. In the case of *B. napus*, about 23% (3/13) genes were detected in this amphidiploid species as compared to the number that, theoretically, could be expected. In this case also, collinearity was observed for about half of the genes (6/13).

**Table 1:** Information of the biotin carboxyl carrier protein (*BCCP*) genes identified in five *Brassica* species^a^, including their genomic locations, protein properties, and subcellular locations

									**Subcellular localization**	
**Gene Name**^**a**^	**Gene Identifier**	**Chromo-some**	**Genomic position**	**Strand**	** CDS**	**Number of AA**	**MW**	**PI**	**WolF PSORT**^**b**^	**mGOASVM**
*B. rapa*										
*BraBCCP1*	*Bra021128*	A01	22,981,012-22,982,979	-	750	276	29.23	8.77	Chlo: 13	Chloroplast
*BraBCCP2*	*Bra023563*	A02	3,944,979-3,946,752	-	831	254	27.10	8.40	Chlo: 12	Chloroplast
*BraBCCP3*	*Bra006305*	A03	2,935,603-2,937,097	+	1539	311	32.99	7.59	Chlo: 10	Chloroplast
*BraBCCP4*	*Bra006352*	A03	3,141,833-3,144,066	-	936	264	27.91	7.54	Chlo: 11	Chloroplast
*BraBCCP5*	*Bra027217*	A05	2,0479,169-2,0481,091	-	795	270	28.62	7.69	Chlo: 12	Chloroplast
*BraBCCP6*	*Bra008694*	A10	13,425,314-13,426,957	-	774	257	27.39	8.40	Chlo: 9	Chloroplast
*B. oleracea*										
*BolBCCP1*	*Bol034727*	C01	33,268,493-33,270,585	-	792	263	27.96	8.27	Chlo: 9	Chloroplast
*BolBCCP2*	*Bol021283*	C02	3,845,325-3,847,265	-	849	282	29.67	9.15	Chlo: 13	Chloroplast
*BolBCCP3*	*Bol034343*	C03	2,767,758-2,769,253	+	771	460	49.44	7.59	Chlo: 10	Chloroplast
*BolBCCP4*	*Bol034400*	C03	3,104,688-3,106,897	-	969	256	27.35	8.96	Chlo: 13	Chloroplast
*BolBCCP5*	*Bol028031*	C03	7,434,514-7,435,380	+	312	322	34.63	8.77	Cyto:8 Chlo: 1	Chloroplast
*BolBCCP6*	*Bol011186*	C05	27,789,227-27,790,999	-	762	256	27.35	8.73	Chlo: 12	Chloroplast
*BolBCCP7*	*Bol030460*	C09	33,382,126-33,383,598	-	747	282	29.83	7.71	Chlo: 10	Chloroplast
*B. nigra*										
*BniBCCP1*	*BniB01g049200.2N*	B01	51,441,670-51,443,749	-	801	266	28.24	8.93	Chlo: 11	Chloroplast
*BniBCCP2*	*BniB02g001110.2N*	B02	686,617-693,316	+	3477	1158	126.11	5.84	Chlo: 9	Mitochondrion
*BniBCCP3*	*BniB02g047380.2N*	B02	50,334,471-50,336,067	-	903	300	32.17	8.20	Chlo: 5, mit:4.5	Chloroplast
*BniBCCP4*	*BniB05g022720.2N*	B05	11,144,486-11,147,592	+	1527	508	55.07	8.39	Chlo: 14	Chloroplast
*BniBCCP5*	*BniB07g054020.2N*	B07	54,106,572-54,108,599	-	795	264	28.23	7.57	Chlo: 9	Chloroplast
*BniBCCP6*	*BniB08g007440.2N*	B08	3,573,114-3,574,646	+	759	252	26.90	6.91	Chlo: 13	Chloroplast
*BniBCCP7*	*BniB08g007850.2N*	B08	3,754,381-3,756,555	-	833	277	29.30	8.44	Chlo: 12	Chloroplast
*B. napus*										
*BnaBCCP1*	*BnaA03g05470D*	A03	2,497,083-2,498,938	+	765	254	26.95	7.65	Chlo: 13	Chloroplast
*BnaBCCP2*	*BnaA03g06000D*	A03	2,721,568-2,724,052	-	828	275	29.21	8.44	Chlo: 12	Chloroplast
*BnaBCCP3*	*BnaA10g18680D*	A10	13,471,949-13,473,830	-	768	255	27.27	8.70	Chlo: 10	Chloroplast
*BnaBCCP4*	*BnaC02g06560D*	C02	3,509,492-3,511,806	+	843	280	29.55	9.15	Chlo: 13	Chloroplast
*BnaBCCP5*	*BnaC03g07000D*	C03	3,331,383-3,333,212	+	771	256	27.33	8.96	Chlo: 10	Chloroplast
*BnaBCCP6*	*BnaC03g07750D*	C03	3,656,512-3,658,874	-	837	278	29.39	6.75	Chlo: 13	Chloroplast
*BnaBCCP7*	*BnaC05g24510D*	C05	18,988,865-18,989,732	-	240	79	8.71	4.29	Chlo: 10	Chloroplast
*BnaBCCP8*	*BnaC06g34180D*	C06	33,750,776-33,751,642	-	318	105	11.64	4.49	Chlo: 9	Chloroplast
*BnaBCCP9*	*BnaC09g42420D*	C09	43,982,680-43,984,535	+	759	252	26.79	6.91	Chlo: 11	Chloroplast
*BnaBCCP10*	*BnaAnng22560D*	*-*	-	+	831	276	29.23	8.77	Chlo: 13	Chloroplast
*B. juncea*										
*BjuBCCP1*	*BjuA005966*	A01	34,734,387-34,736,355	-	750	249	26.55	8.99	Chlo: 10	Chloroplast
*BjuBCCP2*	*BjuA041321 *	A02	3,402,157-3,403,534	-	819	273	28.92	8.77	Chlo: 13	Chloroplast
*BjuBCCP3*	*BjuA041779*	A03	3,230,062-3,231,564	-	765	254	26.99	7.65	Chlo: 12	Chloroplast
*BjuBCCP4*	*BjuA041819*	A03	3,462,118-3,464,771	-	1029	343	37.01	9.21	Chlo: 14	Chloroplast
*BjuBCCP5*	*BjuA020260*	A05	23,641,299-23,643,828	+	959	310	34.55	8.17	Chlo: 8,mito: 4	Chloroplast
*BjuBCCP6*	*BjuA044770 *	A10	14,969,088-14,970,697	-	846	282	30.43	7.71	Chlo: 8,mito: 4	Chloroplast
*BjuBCCP7*	*BjuB025635 *	B01	40,239,002-40,240,483	-	786	262	27.80	8.79	Chlo: 11	Chloroplast
*BjuBCCP8*	*BjuB000235*	B02	45,641,451-45,642,931	-	774	258	27.48	7.02	Mito:7, Chlo: 6	Chloroplast
*BjuBCCP9*	*BjuB035750*	B02	45,675,880-45,677,467	-	798	265	28.22	7.02	Mito:7, Chlo: 6	Chloroplast
*BjuBCCP10*	*BjuB013116 *	B05	16,057,351-16,060,468	+	1341	447	48.35	9.13	Chlo: 11	Chloroplast
*BjuBCCP11*	*BjuB007335*	B07	4,108,276-4,109,783	+	834	245	26.05	5.22	Chlo: 11	Chloroplast
*BjuBCCP12*	*BjuB015095 *	B08	9,808,312-9,810,486	-	735	277	29.30	8.44	Chlo: 12	Chloroplast
*BjuBCCP13*	*BjuB015055 *	B08	9,633,330-96,34,759	+	795	265	28.36	6.17	Chlo: 10	Chloroplast

^a^Bna, Bra, Bol, Bni, and Bju represents the genome data of *B. napus*, *B. rapa*, *B. oleracea*, *B. nigra* and *B. juncea*, respectively

^b^WoLF PSORt predictions: chlo, chloroplast; cyto, cytosol; mit, mitochondria; ext, extracelluar

Analysis of the physiochemical properties of BCCP proteins showed that their length in the three diploid species varied between 252 to 1158 amino acids, and their predicted molecular weights (MWs) and isoelectric point (pI) values varied between 26.90 and 126.11 kDa and 5.84 and 9.15, respectively. In the case of *B. napus*, the length of BCCP proteins varied between 79 and 280 amino acids, their MW between 8.71 and 29.55 kDa, and their pI values between 4.29 and 9.15, where BnaBCCP7 was observed to be the shortest protein with the lowest MW and pI whereas BnaBCCP4 was the longest protein, with the greatest MW and pI values. Among the 13 BCCP proteins in *B. juncea,* the above-mentioned three parameters varied between 245 and 447 amino acids, 26.05 and 48.35 kDa and 5.22 and 9.21 pI, where BjuBCCP10 was the greatest and BjuBCCP11 was the least for these three parameters (Table 1). The predictions of subcellular localization of the BCCP proteins using WolF PSORT analysis indicated that 37 were predicted to be located in the chloroplast, while one BCCP (BolBCCP4) was predicted to be localized to the cytosol and five (BniBCCP3* &* BjuBCCP5, 6, 7,8) were predicted to be targeted to the mitochondria (Table 1). On the other hand, mGOASVM analysis predicted all BCCP proteins to be localized in the chloroplast, except BniBCCP2.

### Phylogeny, gene structure and conserved domain analysis of *Brassica BCCP*s

**Fig. 1:**
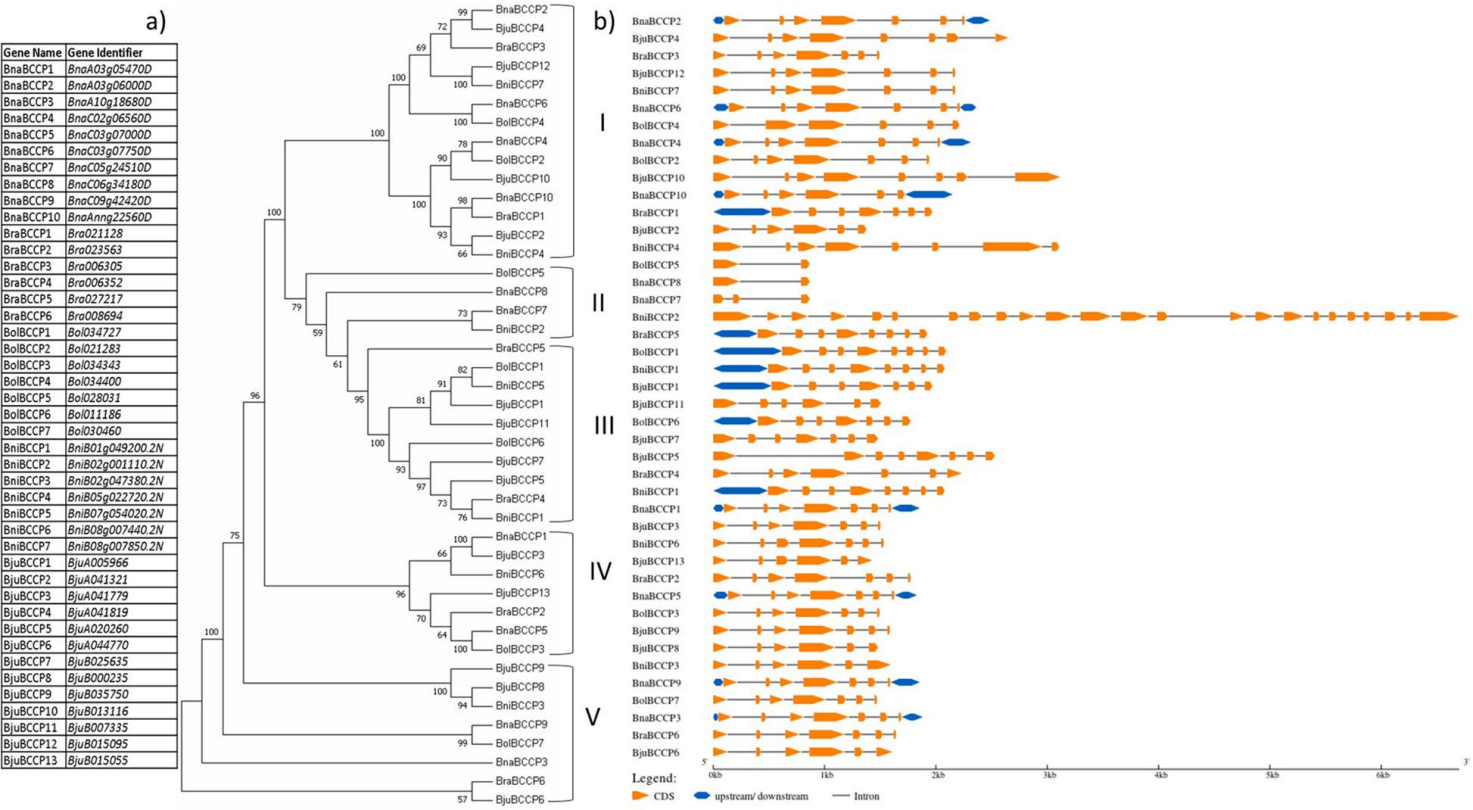
Phylogenetic tree and gene structure of the *BCCP* gene family in *Brassica*. **a** The phylogenetic tree of all proteins in five *Brassica* species (*B. rapa*, *B. oleracea*, *B. nigra*, *B. napus* and *B. juncea*) was developed using MEGA 7. An unrooted tree was developed using the neighbour-joining (NJ) algorithm, the percentage bootstrap values (how often the same branch could be detected when the analysis is repeated) are indicated on the nodes. **b** Organization of exon/intron structures of the *BCCP* genes. Orange boxes represent exons, black lines represent introns and blue boxes indicate untranslated regions (UTR). The length of genes can be estimated by the scale at the bottom

The phylogenetic analysis of the 43 BCCP protein sequences using NJ method resolved them into five classes (I-V) (Fig. 1a), with 14, 4, 10, 7, and 8 members, respectively. Class I, IV and V had representative members from all five *Brassica* species, class II from *B. napus*, *B. nigra* and *B. oleracea* and class III from all four species except *B. napus*. Members of class I, III, IV and V had higher bootstrap values and were included in close clusters, except for BraBCCP5 which branched out from the sub-tree III with a bootstrap value of 79, and BnaBCCP3, BraBCCP6 and BjuBCCP6 which branched out from the sub-tree V (Fig. 1a). Class II had four members with lower bootstrap values (Fig. 1a).

In order to analyse the structure of the 43 *BCCP* genes, we determined the number of exons and introns by comparing the full length CDS and genomic sequences. As shown in Fig. 1b, the class II genes showed the greatest variability in terms of gene length, where *BniBCCP2* was found to be the longest (> 6 kb) among the 43 genes. The other three members of this class had gene lengths < 1 kb. The exon/intron distribution pattern in all classes varied form 6–8 exons (5–7 introns). Members of class II showed greater variability in terms of number of exons, where the *BniBCCP2* sequence contained 24 exons while the *BnaBCCP8* sequence carried 2 exons (Supplementary File 2). Moreover, 15 out of the 43 *BCCP* genes had UTR regions at one or both ends (Fig. 1b).

**Fig. 2:**
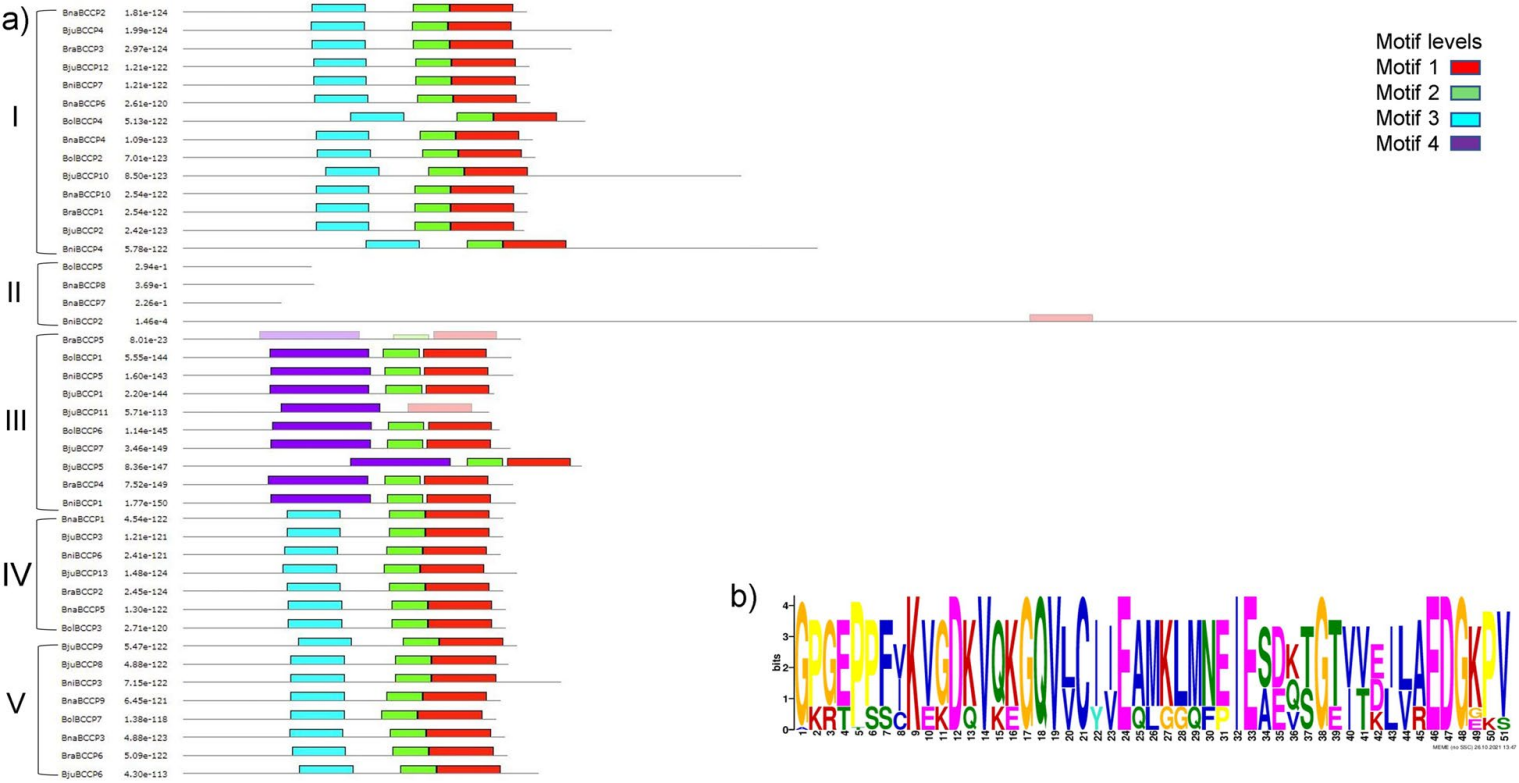
Conserved motif analysis of the *BCCP* genes using MEME v5. **a** Four conserved motifs were identified as represented in different colored boxes while the lengths of the proteins are indicated by black lines. **b** Sequence of motif 1 corresponding to one of the conserved biotinyl domain “CIIEAMKLMNEIE” or “CIVEAMKLMNEIE” or “CYIEQLGGQFPIESDVTGEVVKI”. Note: MEME results don’t show any biotinyl domain for *BraBCCP*5, *BolBCCP*5, *BniBCCP*2, *BnaBCCP*8, *BnaBCCP*7 and *BjuBCCP11* (depicted by absence of red bar in the figure). However, manual inspection showed presence of biotinyl domain in all these members as shown in Supplementary File 2

The BCCP amino acid sequences were further analyzed for the prediction of conserved motifs using MEME suite. A total of four conserved motifs were identified in the 43 *Brassica* BCCPs. Motif 1 was the biotinylation motif, and it was present in 37 members except BraBCCP5, BolBCCP5, BniBCCP2, BnaBCCP8, BnaBCCP7 and BjuBCCP11 (Fig. 2a). However, manual inspection confirmed the presence of biotinylation domains in all these members as shown in Supplementary File 2. Although, motif 2–4 did not belong to any known functional domains based on database searches using InterProScan, motif 2 was primarily present at the C-terminus close to the biotinylation domain while, motifs 3 and 4 were present in the central region. Members of class I, IV and V BCCPs showed a similar arrangement of motifs 3, 2 and 1 from N- to C- terminal of protein, while class III members lacked motif 3 but contained motif 4 instead (Fig. 2a).

### Chromosomal location and gene duplication

**Fig. 3:**
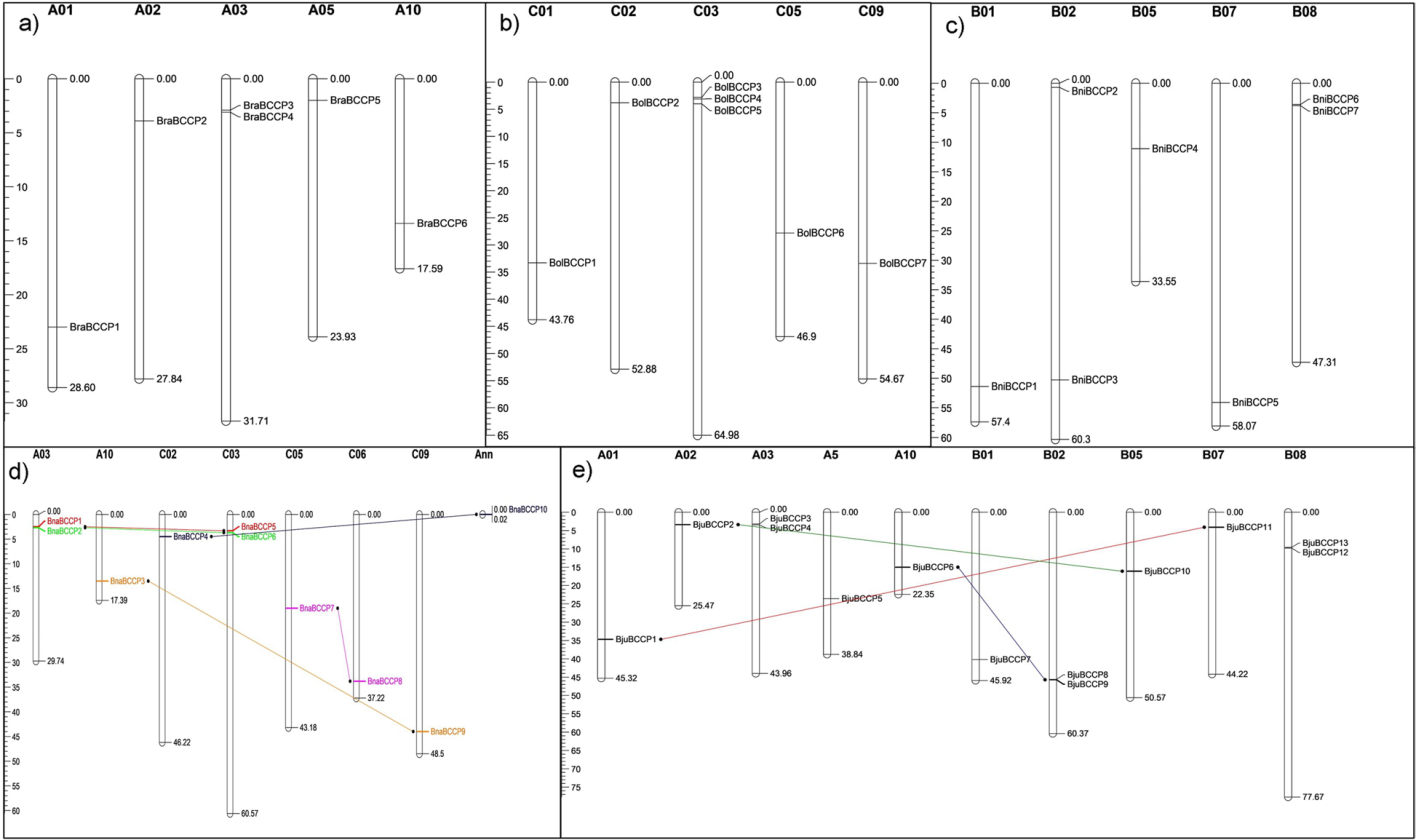
Chromosomal distribution and duplication event coordinates of the *BCCP* genes in five *Brassica* species. Forty-three *BCCP* genes were mapped on different chromosomes in *B. rapa* (**a**), *B. oleracea* (**b**), *B. nigra* (**c**), *B. napus* (**d**) and *B. juncea* (**e**). Only the chromosomes where the *BCCP* genes were mapped are shown. The chromosome number is indicated at the top of each chromosome. The physical position (Mb) of the mapped gene is indicated by a scale (Mb) on the left side of the chromosomes of each species. Genes with segmental duplications are connected by lines. No tandem duplication was found in any of the five *Brassica* species

We investigated the distribution of the 43 *BCCP* genes in the five *Brassica* species based on their chromosomal location in the BRAD database and constructed physical maps for these species (Fig. 3). In *B. napus*, one *BCCP* gene was detected to be localized on each of the chromosomes A10, C02, C05, C06 and C09 while each of A03 and C03 harbored two *BCCP* genes (Fig. 3d). *BnaBCCP10* appeared to be anchored on an unmapped scaffold and, therefore, we could not assign this to any of the sub-genomes. In the case of *B. rapa*, one *BCCP* gene was found on chromosome A01, A02, A05 and A10, while chromosome A03 contained two *BCCP* genes (Fig. 3a). Chromosome C03 of *B. oleracea* harbored the maximum number of *BCCP* genes (three), while each of chromosomes C01, C02, C05 and C09 carried one gene (Fig. 3b). The *B. nigra* genome contains seven *BCCP* genes distributed across five chromosomes (Fig. 3c). Among the 13 *BCCP* genes detected in *B. juncea*, six were assigned to the A sub-genome and seven to the B sub-genome (Fig. 3e). It can, therefore, be determined from our results that a few chromosomes of each of the three *Brassica* genomes apparently lack *BCCP* genes; however, the 43 genes that we identified in this study were almost uniformly distributed throughout the rest of the chromosomes. In addition, we observed that most of the *BCCP* genes in the allotetraploid species are located at an approximate position of the chromosome as observed in the diploid species. For instance, *BraBCCP3* and *BraBCCP4* (2.9 and 3.1Mbp) of *B. rapa*, *BnaBCCP1* and *BnaBCCP2* (2.5 and 2.7 Mbp) of *B. napus*, and *BjuBCCP3* and *BjuBCCP4* (3.2 and 3.4 Mbp) of *B. juncea* were located on chromosome A03 at about the same position (Fig. 3; Table 1). Similarly, *BraBCCP6* and *BnaBCCP3* were located at about 13.14 Mbp, while the *BjuBCCP6* was located at 15 Mbp of chromosome A10. Furthermore, *BraBCCP2* and *BjuBCCP2* were observed to be located on chromosome A02 at 3.9 and 3.4 Mbp, respectively. In the case of *BCCP* genes located on the C-genome, *BolBCCP2* and *BnaBCCP4* in chromosome C02 were located at 3.8 and 3.5 Mbp, respectively. There were also instances where the *BCCP* gene was either missing in the allotetraploid species or the position of the gene was different when compared with the diploid species. For instance, chromosome B02 of *B. nigra* carried two *BCCP* genes at 0.68 Mbp (*BniBCCP2*) and 53.3 Mbp (*BniBCCP3*), whereas B02 of *B. juncea* carried the *BjuBCCP8* and *BjuBCCP9* at 45.64 and 45.68 Mbp, respectively.

We also investigated the gene duplication events of the *BCCP* to understand their roles in genome expansion and re-alignment. Based on alignment of the sequence length and similarity of the aligned regions, no tandem duplication events were observed in any of the five *Brassica* species. To identify segmental duplication events, we used the following criteria: alignment coverage > 80% of the two aligned genes, and identity of the genes > 80%. In the diploid *Brassica* species, no segmental duplication events could be detected, while the allotetraploids *B. napus* and *B. juncea* carried four and three gene pairs exhibiting segmental duplication (Fig. 3). Among them, two gene pairs, *BnaBCCP*3/*BnaBCCP*9, and *BjuBCCP*6/*BjuBCCP*9, belonged to class V and two gene pairs, *BnaBCCP4*/*BnaBCCP10*, and *BjuBCCP*2/*BjuBCCP10*, belonged to class I (Fig. 1). All the duplicated gene pairs were located on different chromosomes, suggesting all of them were, indeed, resulted from segmental duplication events. Thus, the results from this study demonstrated the occurrence of segmental gene duplication events in the expansion of this gene family in the allotetraploid *Brassica* species.

### Orthologous relationship of the *BCCP* genes of the *Brassica* species

To reveal the orthologous relationships of the *BCCP* genes between the five *Brassica* species, their gene sequences were used to construct 10 unrooted phylogenetic trees (Supplementary Fig. 1). The results indicated that there were 29 pairs of orthologous genes among the five species since they were in terminal branches with high bootstrap values (> 85). Among them, the diploid species had 14 orthologous gene pairs i.e., five in each of *B. rapa* and *B. oleracea*, five in each of *B. oleracea* and *B. nigra* and four in each of *B. rapa* and *B. nigra*. Four orthologous gene pairs were detected between the two amphidiploid species (*B. napus* and *B. juncea*). On the other hand, no orthologous gene pairs were identified between *B. oleracea* and *B. juncea*; two orthologous gene pairs between *B. napus* and *B. nigra*, three between *B. napus* and *B. rapa*, four between *B. napus* and *B. oleracea*, three between *B. juncea* and *B. nigra* and one between *B. rapa* and *B. juncea* were identified.

### Syntenic relationship of *BCCP*s in *A. thaliana* and five *Brassica* species

Based on the extent of gene retention or loss, the *Brassica* genomes can be partitioned into three sub-genomes, namely LF (least fractionated), MF-I (moderately fractionated), and MF-II (most fractionated) [21, 41]. For each *Brassica BCCP* gene, we identified its syntenic paralog in its respective sub genomes as well as its orthologs in *A. thaliana* from the BRAD database. The syntenic relationship between the *BCCP* genes of *B. napus*, *B. rapa*, *B. oleracea*, *B. juncea*, *B. nigra* and *A. thaliana* are summarized in Supplementary File 3. For 90.6% (39/43) of the *Brassica BCCP* genes, their orthologs could be found in *A. thaliana*, while orthologs could not be detected for the remaining (9.4%; 4/43) genes (*BnaBCCP7*, *BnaBCCP10*, *BolBCCP*5 and *BjuBCCP8*). Among the 10 *B. napus BCCP* genes, one was located on the LF (A genome), two on the MF1 (A genome), one on the LF (C genome), two on the MF1 (C genome) and one on the MF2 (C genome) sub-genomes. Similarly, for the 13 *BjuBCCP* genes, two were located on the LF (A genome), three on the MF1 (A genome), one on the MF2 (A genome), two on the LF (C genome), three on the MF1 (C genome) and one genes on the MF2 (C genome) sub-genomes. For each of the *A. thaliana BCCP* gene, three copies were expected in each of the *Brassica* genomes resulting from a whole-genome triplication (WGT) event during their evolution [42]. Interestingly, all three diploid *Brassica* species i.e. *B. rapa*, *B. oleracea* and *B. nigra* carried two gene copies for each of the *A. thaliana BCCP* gene indicating gene loss might have occurred following the WGT event. For instance, *AtBCCP1* was the ortholog of the *B. rapa* genes *BraBCCP2* and *BraBCCP4*; *AtBCCP2* was the ortholog of *BraBCCP3* and *BraBCCP6* and, *AtBCCP2*-like was ortholog of *BraBCCP1* and *BraBCCP5* (Supplementary File 3). Each of the A and B genomes of the allotetraploid species *B. juncea* carried two copies of each of the *A. thaliana BCCP* gene. In the case of *B. napus*, the A genome carried one or two copies and the C genome carried two copies of the *A. thaliana BCCP* gene. This indicates that loss or expansion of the *BCCP* gene family occurred in *B. napus*.

To gain insights into the selective pressure on the *BCCP* genes, the Ka, Ks and Ka/Ks values, as well as divergence time of the genes were calculated for different orthologous *BCCP* gene pairs between *A. thaliana* and *Brassica* (Supplementary File 4). The Ka/Ks ratios varied between 0.600 and 1.580 for *B. napus*, 0.534 and 1.117 for *B. rapa*, 0.436 and 1.282 for *B. oleracea*, 0.561 and 1.467 for *B. nigra*, and 0.512 and 1.032 for *B. juncea*. Seven *BCCP* gene orthologs of *AtBCCP1* and 11 genes orthologs of *AtBCCP2* had Ka/Ks ratio greater than 1 in the diploid and amphidiploid species, suggesting these genes have experienced positive selection (beneficial alleles increasing in prevalence), while all other gene pairs have Ka/Ks ratios less than 1, implying purifying selection (detrimental alleles eliminated) played a role during the process of species evolution. The Ka/Ks ratios for the duplicated gene pairs ranged between 0.550 and 1.442 again suggesting the involvement of a purifying and positive selection during species evolution.

### Comparative phylogeny of the *Brassica BCCP* genes with other plant *BCCP*s

**Fig. 4:**
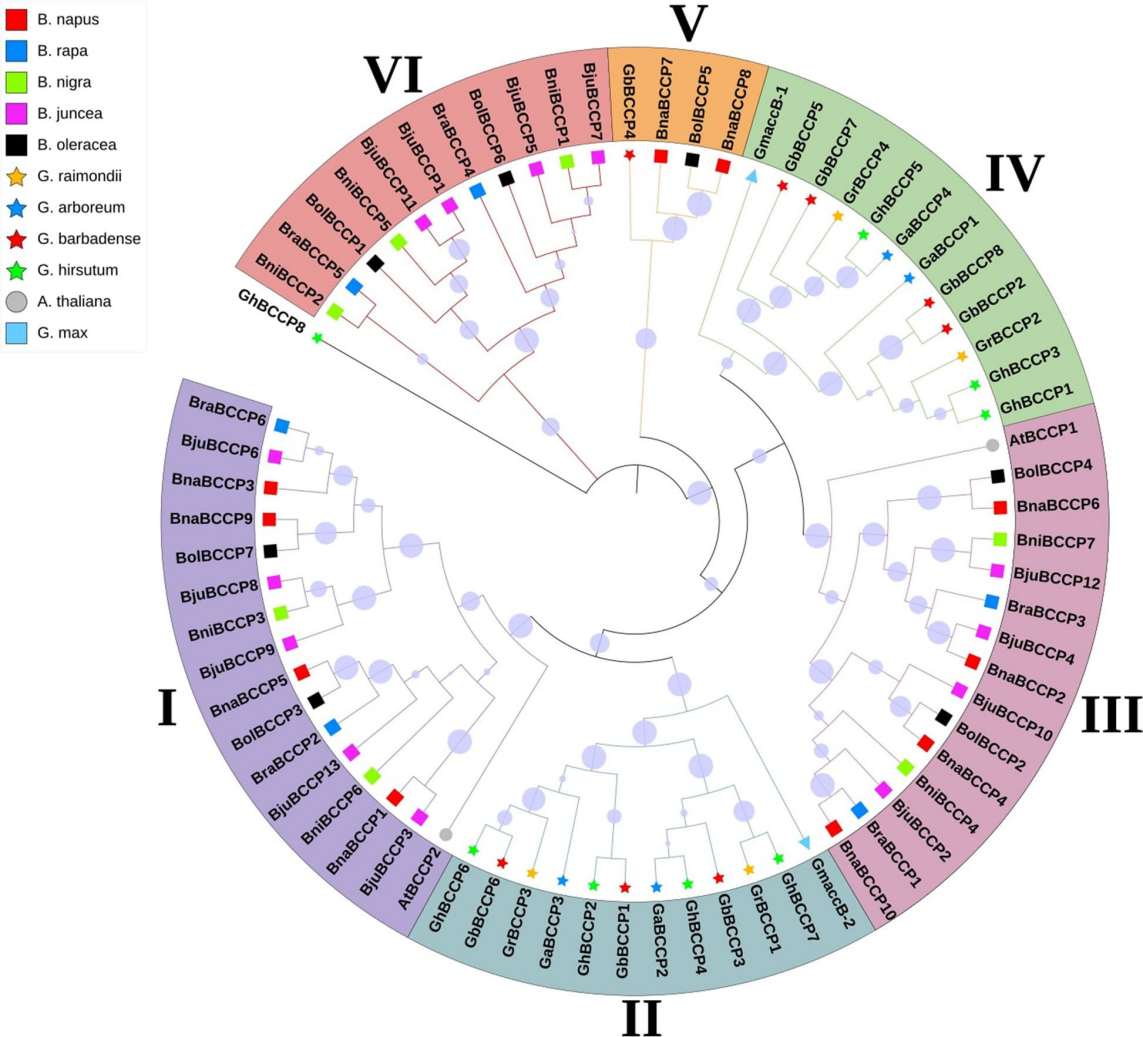
Phylogenetic tree of the BCCP proteins in *Brassica* and other plants. Unrooted phylogenetic tree was generated using Neighbor-Joining (NJ) method using two *Arabidopsis* (names starting with At), two *Glycine max* (names staring with Gm), 24 *Gossypium* (names starting with Ga or Gb or Gh or Gr) and 43 *Brassica* (names starting with B) BCCP proteins. Bigger circle on the internal nodes indicates higher percentage of bootstrap values. BCCP proteins from different species are marked with different colored shapes. The six groups (I-VI) of BCCP proteins are showed with different colors

To understand the evolutionary pattern of the *Brassica BCCP* gene family in relation to other plant family genes, a NJ phylogenetic tree was constructed by using the *BCCP* protein sequences of the five *Brassica* species, *A. thaliana*, *G. max* and *Gossypium* (Fig. 4). The *BCCP*s broadly grouped into six clusters supported by significant bootstrap values. All the *Brassica* and *A. thaliana BCCP*s were grouped in four clusters, namely, class I, III, V and VI, suggesting a close relationship between them. The *Gossypium BCCP*s formed their own two clusters i.e., class II and IV, while the *GbBCCP4* clustered with three *Brassica BCCP*s in class V, and *GhBCCP8* did not cluster into any classes. The two *G. max BCCP* proteins clustered in class IV (*GmaccB*-*1*) and II (*GmaccB*-*2*). These results suggest that the *BCCP*s have possibly undergone independent sequence diversification in different organisms that were investigated.

### *Cis*-regulatory element analysis and miRNA prediction

To elucidate the possible regulation of the expression of *Brassica*e *BCCP* genes in response to abiotic and biotic stress, the promoter sequences (2 Kb upstream region) of the 43 *BCCP* genes were analyzed using PLANTCARE. Twelve types of stress- and hormone related *cis*-acting regulatory elements were detected in single or multiple copies, and this included AuxRR-core, TGA element (auxin responsive), GARE-motif and TATC-box (Giberellin responsive), ABRE (responsive to Abscisic acid), TGACG and CGTCA motif (responsive to MeJA), TCA-element (SA responsive), LTR (low temperature responsive), MBS (MYB element for drought responsive), and TC-rich repeats and WUN-motif (defense and stress response) (Supplementary File 5). The *BCCP* genes, viz. *BraBCCP3*, *BolBCCP3*, *BniBCCP6*, *BnaBCCP1*, *BnaBCCP7* and *BjuBCCP11* of the five *Brassica* species had 31, 30, 22 19, 19 and 23, *cis* elements, respectively. No cis elements were detected in *BolBCCP*6. Notably, 41 *Brassica BCCP* genes (95.4%) had ABA-responsive elements in the promoter regions implying their possible role in mediating responses to ABA.

In order to determine whether the *BCCP* genes are regulated by miRNAs, we analyzed them for the presence of miRNA target sites in the five *Brassica* species using psRNA-Target web server with default parameters. The psRNA-Target webserver lists the miRNAs for *B. napus*, *B. rapa* and *B. oleracea*, but not for *B. nigra* and *B. juncea*. Therefore, we conducted a literature search for the miRNAs reported in the available literature for *B. nigra* and *B. juncea*. No literature could be found for miRNAs identified in *B. nigra*, but we found two papers reporting miRNAome of *B. juncea* [43, 44]. No miRNAs could be detected for the *B. oleracea BCCP*s when we used 11 *B. oleracea* miRNAs submitted to psRNA-target. Therefore, we extracted a list of *B. oleracea* miRNAs from Lukasik et al*.* [45] and used them for miRNA target site prediction. Alignment of the *BCCP* transcripts with the miRNA sequences identified three, seven, four and six miRNAs in *B. napus*, *B. rapa*, *B. oleracea* and *B. juncea*, respectively (Supplementary File 6). In *B. napus*, *BnaBCCP*3 was predicted to be targeted by a, b, and c members of miR390. Four members of *B. rapa BCCP* family were predicted to be targeted by miR390-5p (*BraBCCP*6), miR398-5p (*BraBCCP*5), miR5722 (*BraBCCP*3), and miR5720, miR9560a-5p, miR9560b-5p and miR9563b-3p (*BraBCCP*4). The *BolBCCP*2 and *BolBCCP*6 were predicted to be regulated by miR5021 family, i.e., miR5021a/f/j and miR5021f, respectively. Six *BjuBCCP*s were predicted to be targeted by miR5015b, miR5021 and miR5658.

### Expression patters of the *BnaBCCP* genes under biotic stresses

#### *Plasmodiophora brassicae* infection

**Fig. 5:**
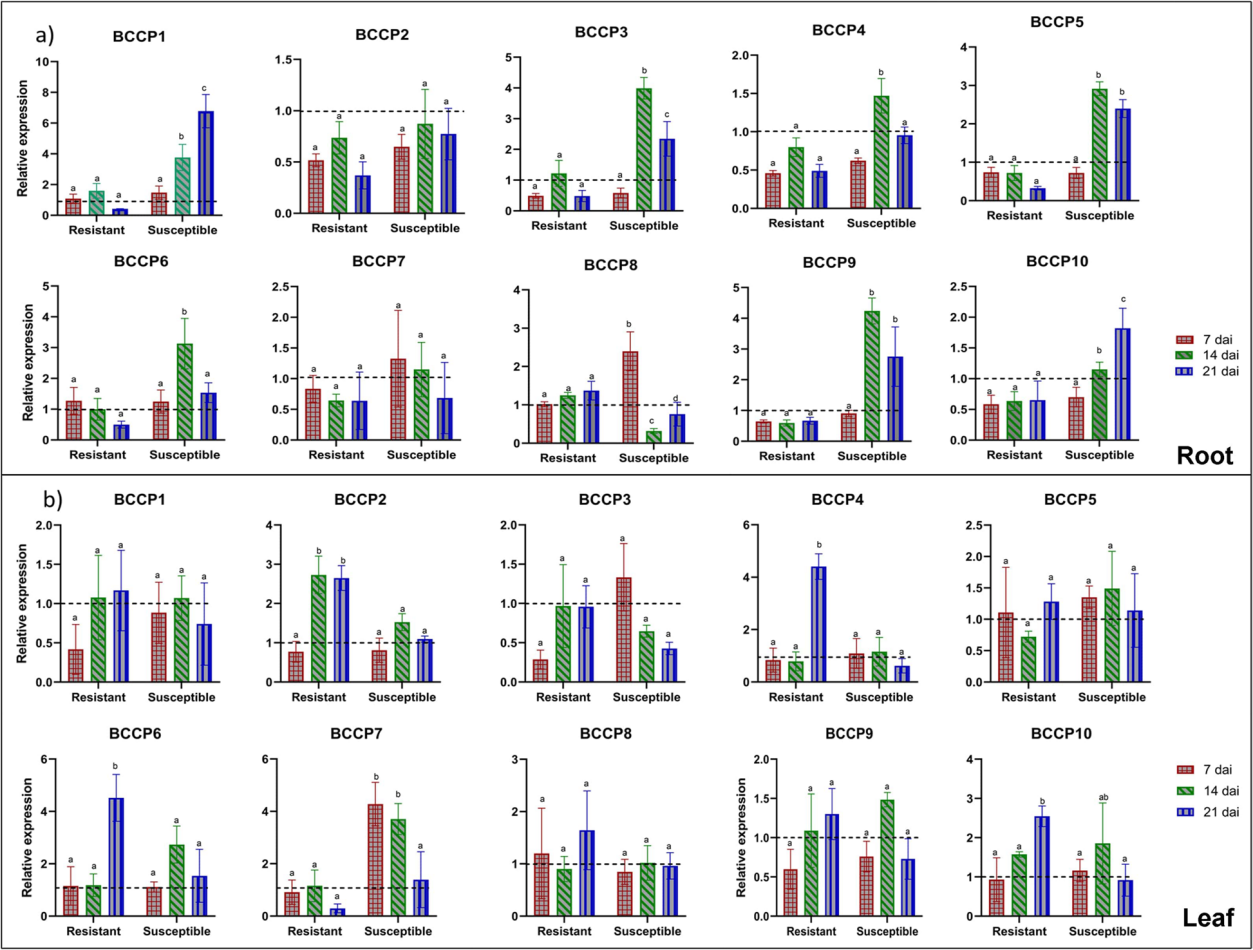
Expression analysis of the *BnaBCCP* genes in resistant and susceptible plants upon *Plasmodiophora brassica*e infection. Expression patterns of 10 *BnaBCCP* genes in **a**) roots and **b**) leaves of infected resistant and susceptible bulks at 7, 14 and 21 days after inoculation) dai. The time points are represented by x-axis and scale of relative expression is shown by y-axis. The expression level was normalized to control of each time point and indicated by dotted line at expression level = 1. The housekeeping gene *UBC9* was used an internal control. Error bars indicate means of three biological replicates ± standard errors (SEs). Different letters indicate significant differences in mean values (*P* < 0.05, Tukey method)

To explore the differential response of the 10 *B. napus BCCP* genes to pathogen infection, qRT-PCR of these genes was performed to determine their relative expression in root and leaf samples of two contrasting bulks (resistant and susceptible) at different time points post-inoculation with *P. brassicae*. The qRT-PCR results revealed that expression of eight genes was induced in response to infection in roots of the susceptible bulk at one to all three time points, while the remaining two genes (*BnaBCCP2* and *BnaBCCP7*) showed no significant change in their transcript accumulation (Fig. 5). Out of the eight genes, *BnaBCCP1* showed a gradual increase in transcript abundance over the course of infection and showed a maximum expression of 6.7-fold at 21 dai (days after infection), while the expression of five genes (*BnaBCCP3*, *4*, *5*, *6* & *9*) peaked at 14 dai, and the expression of two genes *BnaBCCP8* and *BnaBCCP10* was highest at 7 dai and 21 dai, respectively. All 10 *BnaBCCP* genes showed no significant change in expression over course of infection in roots of the resistant bulk (Fig. 5a). In the leaf tissues of the resistant bulk, two *BCCP* genes (*BnaBCCP4* and *BnaBCCP6*) showed an increased expression (4.4 and 4.5-folds) at 21 dai, while the expression of *BnaBCCP2* was significantly upregulated at both 14 and 21 dai, and *BnaBCCP10* was significantly upregulated at 21 dai (Fig. 5b). In case of the susceptible bulk, expression of only one gene (*BnaBCCP7*) showed a significant change at 7 and 14 dai.

#### *Sclerotinia sclerotium* infection

**Fig. 6:**
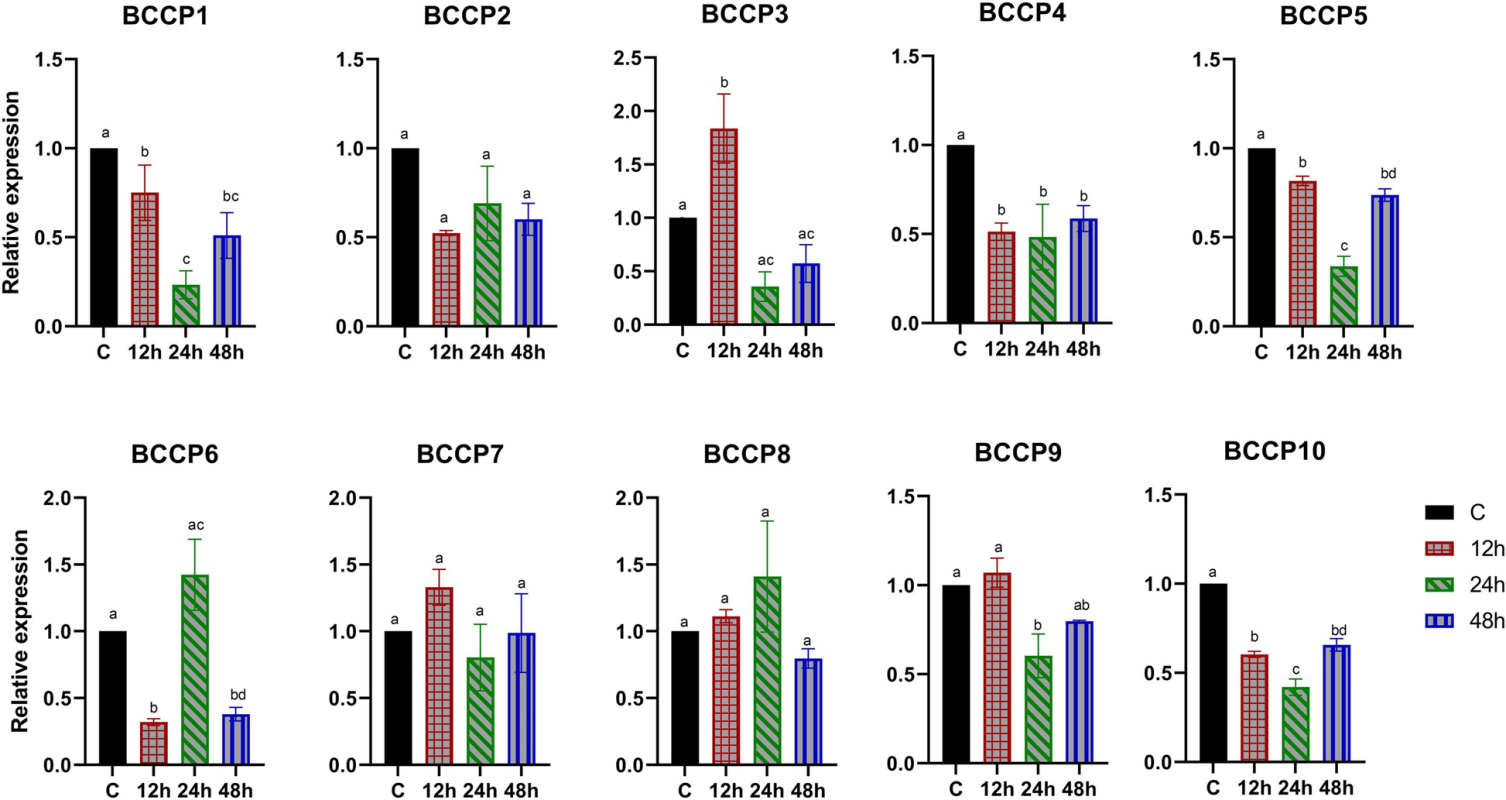
Expression analysis of the *BnaBCCP* genes in DH12075 upon *Sclerotinia sclerotium* infection. Expression patterns of the *BnaBCCP* genes in leaves of the *Brassica napus* line DH12075 during a compatible interaction after 12, 24 and 48 h of *S. sclerotium* infection. The time points are represented by x-axis and scale of relative expression is shown by y-axis. The expression level was normalized to control of each time point with expression level = 1. The housekeeping gene *UBC9* was used an internal control. Error bars indicate means of three biological replicates ± standard errors (SEs). Different letters indicate significant differences in mean values (*P* < 0.05, Tukey method)

In case of sclerotinia infection, expression of four members (*BnaBCCP1*, 4, *5* & *10*) showed a significant decrease, while expression of *BnaBCCP2*, *7* & *8* showed no significant change when compared with control at each time point (12, 24 and 48 hai (hours after infection)). *BnaBCCP3* was the only gene showing an increased expression of 1.8-fold at 12 hai followed by a significant decrease in expression at 24 and 48 hai (Fig. 6).

### Expression profile of the *BnaBCCP* genes under abiotic stresses

#### Cold stress

**Fig. 7:**
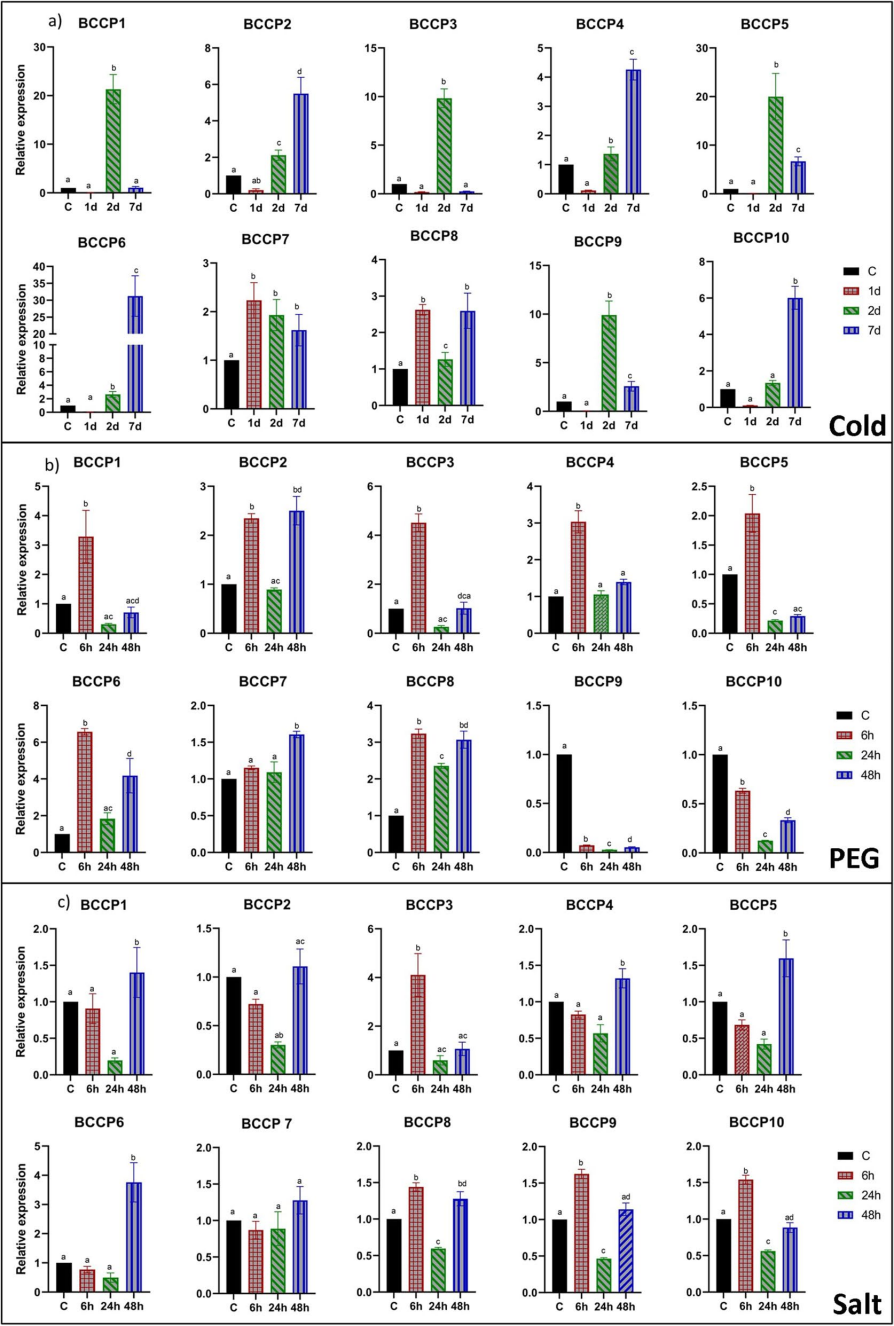
Expression analysis of the *BnaBCCP* genes in DH12075 after different abiotic stresses. Expression patterns of the genes in leaf samples collected after 6, 24 and 48 h of **a**) cold, **b**) PEG, and **c**) salt stress. The time points are represented by x-axis and the scale for relative expression of the genes is shown by y-axis. The expression level was normalized to control of each time point. The housekeeping gene *UBC9* was used an internal control. Error bars indicate means of three biological replicates ± standard errors (SEs). Different letters indicate significant differences in mean values (*P* < 0.05, Tukey method)

To identify the *B. napus BCCP* genes involved in plant response to cold stress, 3-week-old seedlings were exposed to cold stress for up to 7 days. One-day of cold exposure did not change expression of eight *BnaBCCP* genes, except for *BnaBCCP7* and *BnaBCCP8* where a 2.5- and 2.6-fold increase was observed (Fig. 7a). After 2 days of cold exposure, > twofold increase was observed for six members i.e., *BnaBCCP1*, *2*, *3*, *5*, *6* & *9*, while seven members showed a > twofold increase 7 days after cold exposure. Among them, expression levels of *BnaBCCP1*, *3*, *5*, *7 & 9* significantly decreased after 7 days when compared to the expression level at 2 days after cold exposure. For four genes (*BnaBCCP2*, *4*, *6* & *10*), transcript accumulation followed a similar trend of continuous increase up to 7 days of stress. Of the 10 *BCCP* genes, *BnaBCCP6* showed the highest transcript accumulation (31.2-fold) 7 days after cold stress.

#### Drought and salinity stress

To gain some information about the response of the *BnaBCCP* genes to drought stress (20% (w/v) PEG8000)), we examined their expression level in leaves of 3-week-old seedlings. The results showed that expression of seven *BnaBCCP* (*BnaBCCP1*, *2*, *3*, *4*, *5*, *6* & *8*) genes was significantly increased at 6 h after PEG treatment, where the expression remained significantly higher than the control at 48 h for *BnaBCCP*2, 6 & 8. On the other hand, the expression of *BnaBCCP9* & *10* was significantly downregulated at all time points due to the PEG treatment (Fig. 7b).

Salt stress on 3-week-old seedlings did not produce any significant change in expression of *BnaBCCP1*, *2*, *4*, *5* & *6* at 6 and 24 h; however, they showed a significantly greater level of expression at 48 h after the treatment (Fig. 7c). Salt stress for a short period of time (6 h) resulted an increased expression for *BnaBCCP3, 8*, *9* & *10*; however, their transcript levels at 48 h dropped similar to control tissues in most of the cases. The greatest expression was observed for *BnaBCCP3* and *BnaBCCP6* at 6 h (4.1-fold increase) and 48 h (3.8-fold increase) after salinity stress, respectively (Fig. 7c).

### Differential expression of the *BnaBCCP* genes in response to treatment with phytohormones

**Fig. 8:**
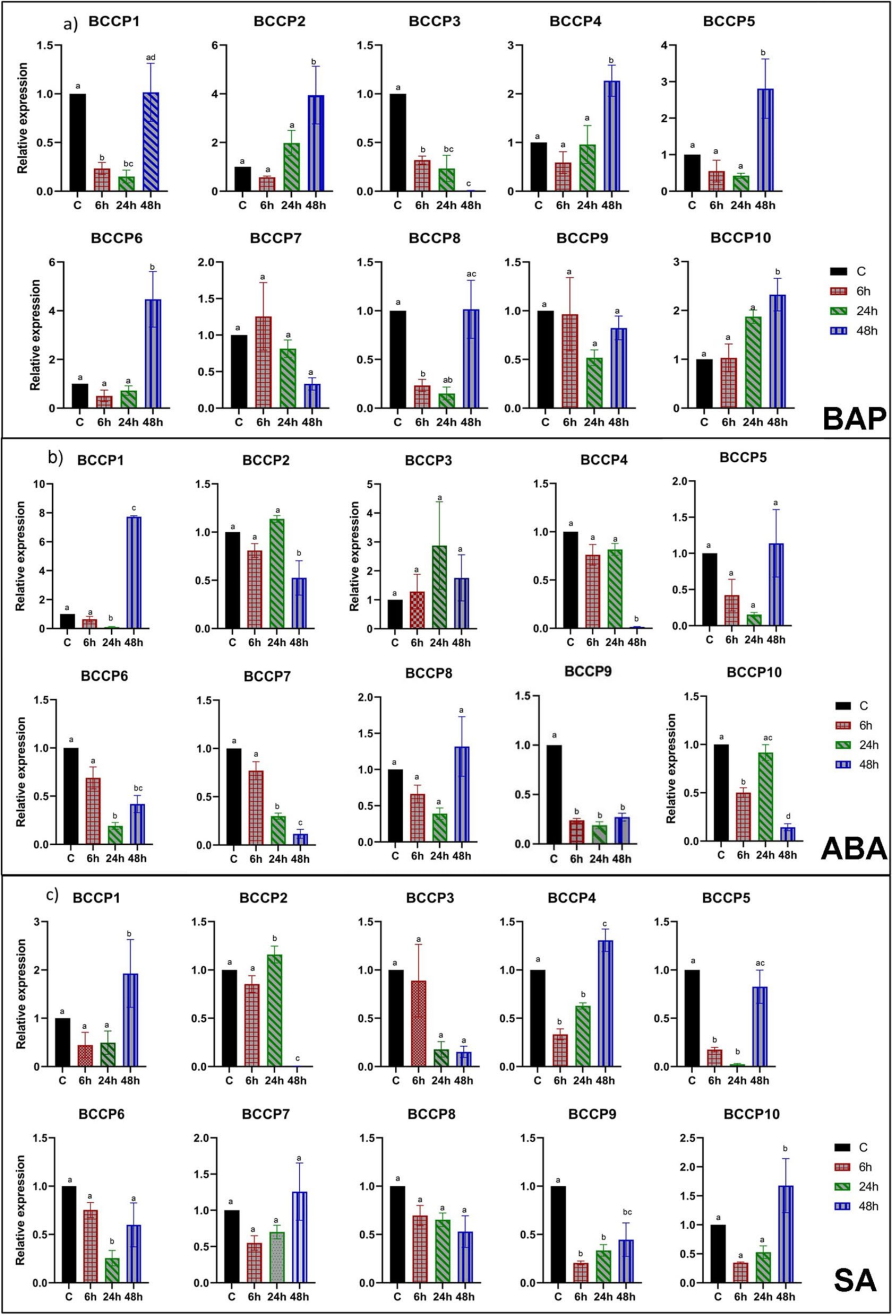
Expression analysis of the *BnaBCCP* genes in DH12075 after hormonal treatments. Expression patterns of the genes in leaf samples collected after 6, 24 and 48 h of **a**) BAP, **b**) ABA, and **c**) SA treatments. The time points are represented by x-axis and the scale of relative expression is shown by y-axis. The expression level was normalized to control of each time point. The housekeeping gene *UBC9* was used an internal control. Error bars indicate means of three biological replicates ± standard errors (SEs). Different letters indicate significant differences in mean values (*P* < 0.05, Tukey method)

In case of BAP treatment, five genes (*BCCP2*, *4*, *5*, *6* & *10*) showed a significant increased expression at 48 h after exposure as compared to control as well as their expression at 6 and 24 h time points (Fig. 8a). Expression of the *BnaBCCP1*, *3* & *8* decreased significantly at 6 and 24 h after the treatment; however, the expression of *BnaBCCP1* and *BnaBCCP8* returned to control levels (= 1), while the expression of *BnaBCCP3* further decreased at 48 h after the treatment. No significant difference in expression was observed in the case of *BnaBCCP7* & *9*.

ABA treatment for a short period of time (6 h) did not change the levels of expression for eight of the 10 genes with the exception of *BnaBCCP9* and *BnaBCCP10* (Fig. 8b). However, 24 or 48 h treatment significantly reduced the expression of *BnaBCCP6*, *7* & *9*. In contrast, expression of *BnaBCCP1* was significantly increased at 48 h; in fact, this gene exhibited the greatest change in expression (increased 7.7-fold). In case of SA treatment, significantly increased expression at 48 h was observed in the case of *BnaBCCP1, 4* & *10*, and decreased expression for *BnaBCCP2* and *BnaBCCP9* (Fig. 8c).

## Discussion

*Brassica* oilseed crops are the second largest source of vegetable oil, after soybean, in the world (FAO 2022), and this includes *B. rapa*, *B. juncea*, *B. napus*, and *B. carinata*. Among these, *B. napus* is the largest both with respect to acreage and production. *B. napus* yields are significantly affected by various biotic stresses, such as insect pests and disease, and abiotic stresses, such as cold, drought and salinity. Therefore, development of cultivars resistant to biotic and abiotic stresses is important for the sustainable production of this crop.

To date, most of the crop improvements in *Brassica* have been achieved through conventional breeding. In the recent years, the release of *Brassica* genome sequences [e.g. 21, 24] provided researchers and breeders a snapshot of the genomes and a suite of putative genes for different crop traits. The genome sequences have been used by different researchers to characterize some of the genes, such as *Crr1a* (for resistance to clubroot disease) in *B. rapa* [46], *ALCATRAZ* (*ALC*) (involved in seed shattering from mature fruits) in *B. napus* [47] and glucosinolate transporters (GTRs) in *B. rapa* and *B. juncea* [48]. The biotin carboxyl carrier protein (BCCP) is known to play an important role in fatty acid biosynthesis and lipid metabolism in plants [11, 12], and fatty acids and lipids play an important role in plant growth and development [49] as well in mediating abiotic stress responses, including cold tolerance [49] and during biotic stresses such as *P. brassicae* infection [49]. Therefore, the knowledge about the roles of *BCCP* genes in mediating resistance to biotic and abiotic stresses in canola may benefit *Brassica* crop breeders to devise rational approaches to improve this crop.

In the present study, we identified 43 *BCCP* genes in five *Brassica* species containing the biotinylation domain. The sequence of these proteins varied in their lengths to some extent in these species, where the greatest variation was observed to be in those *BCCP* genes located on B genome of *B. nigra* (759 to 3477 amino acids) as well as of *B. juncea* (735 to 1341 amino acids). In contrast, the length of *A. thaliana* BCCP proteins, such as AtBCCP1 and AtBCCP2 is very similar (280 and 250 amino acids), while in cotton it varied from 57 to 515 AA [10]. In our study, we found the length of identified sequences varied between 79 and 1158 amino acids residues signifying greater complexity of BCCP proteins in *Brassica* species.

Majority of the BCCP proteins were predicted to be localized in the chloroplast, confirming their role in fatty acid biosynthesis within plastids [16]. The prediction of the sequence motifs showed that all BCCP proteins contained a biotinylation domain at their C-terminal end. In addition to the previously reported CIIEAMKLMNEIE or CIVEAMKLMNEIE domains in other species such as *A. thaliana*, *A. moluccana* [9] and cotton [10], we identified a new domain “CYIEQLGGQFPIESDVTGEVVKI” (amino acids position 21 to 39; Fig. 2b) in six members of *B. rapa*, *B. oleracea*, *B. nigra* and *B. juncea* (*BraBCCP1, BraBCCP5*, *BolBCCP1*, *BniBCCP1*, *BniBCCP5* and *BjuBCCP11*). All the three domains can be categorized under Pfam domain, PF00364, which is known to contain a conserved lysine residue that is covalently linked to biotin or lipoic acid. Biotin plays an role in the catalysis of carboxyl transfer reactions and is covalently attached via an amide bond to a lysine residue in enzymes such as ACCase [50]. No members of *B. napus* BCCPs were found to contain the new domain and had either the CIIEAMKLMNEIE (*BnaBCCP1*, *3*, *5*, *7*, *8* & *9*) or CIVEAMKLMNEIE (*BnaBCCP*2, *4*, *6* & *10*) at the C-terminal both of which contain the conserved lysine (K) for the covalent attachment of biotin.

Exon/intron structural divergence plays an important role in the evolution of multiple gene families. Ren et al*.* [51] reported that, in rice and *A. thaliana*, the highly expressed genes carry longer as well as a greater number of introns and generate larger primary transcripts than the genes expressed at a low level, i.e. the plant genes which express at a greater level tend to be less compact than the genes expressed at a lower level. They found 5.5 and 5.9 introns in the highly expressed genes and 3.8 and 3.6 introns in the low expressed genes in *A. thaliana* and rice, respectively. In this study, we observed that 90% (39/43) of the *BCCP* genes belonging to classes I, III to V had 5 to 7 introns, while three members of class II had 1 to 2 introns only (Fig. 1). Cui et al*.* [10] also reported about 5.7 introns in cotton *BCCP* genes. Our observations suggest that the *BCCP* genes belong to the class of highly expressed genes and, being a major constituent of the ACCase enzyme, its expression is required at all stages of plant growth and development. Thus, most members in the same phylogenetic group had similar motif composition and exon/intron arrangements supporting the phylogenetic classification.

It has been extensively reported that the *Arabidopsis* and *Brassica* genomes evolved from a common ancestor where the *Brassica* genomes experienced a genome triplication event [21, 52, 53]. *Arabidopsis thaliana* carries only two *BCCP* genes [12] which apparently have resulted from gene/genome duplication prior to split of the *Arabidopsis* and *Brassica* lineages about 14.5 to 20.4 MYA (million years ago) [54]. Considering the number of *BCCP* gene copies in *A. thaliana* each of the three *Brassica* genomes, A, B and C, expected to carry six *BCCP* genes. We found six *BCCP* genes in *B. rapa*, seven in *B. nigra* and seven in *B. oleracea*, which is about 3 × number of the *BCCP* genes of *A. thaliana*. The retention of the *BCCP* genes in the three *Brassica* genomes agrees with the gene balance hypothesis proposed by different researchers [55, 56]. This hypothesis states that, the genes whose products participate in signal transduction networks, transcription or in macromolecular complexes, are more likely to be preferentially retained, avoiding imbalances associated with loss of a functional copy [55, 56]. As stated above, *BCCP* is a major constituent of the ACCase enzyme and, therefore, its expression is required at all stages of plant growth.

We also found slight differences in the number of *BCCP* genes in the three *Brassica* genomes. It has been extensively reported that the three *Brassica* genomes evolved from a common ancestor through chromosome fusions/fissions [52], where at least 16 gross chromosomal rearrangements differentiated the A and C genomes during their divergence from the ancestor [57]. This might be one of the reasons for the slight difference in the number of *BCCP* genes in the three diploid genomes (six in *B. rapa* vs. seven in *B. nigra*/*B. oleracea*).

In the case of the two amphidiploid species, the number of *BCCP* genes that we found in *B. juncea* is exactly the sum of the number of genes of its two progenitor species (*B. rapa* and *B. nigra*). In contrast, *B. napus* carried two to three fewer number of genes than the number could be expected based on the number of genes found in *A. thaliana* or in its two progenitor species. The loss of genes seems to have specifically occurred in its A genome. It is widely accepted that *B. napus* evolved from *B. rapa* and *B. oleracea* through interspecific hybridization between these two species. Several researchers [57–59] have reported that genome changes, including loss of DNA fragments can occur in the newly formed allopolyploid species *B. napus*. This might be a reason behind the loss of *BCCP* genes in *B. napus*.

Segmental gene duplication events have been reported to have contributed to the realignment and expansion of organism’s genome [60]. Duplication of genes, such as zinc finger [61], RING finger [62], late embryogenesis abundant (LEA) gene family [63], Receptor-like kinase (RLK) [64], and Xyloglucan endotransglucosylase/hydrolase genes (XTHs) [65] belonging to other gene families have been reported in *Brassica*. We found that seven *BCCP* gene pairs (four in *B. napus* and three in *B. juncea*) were preferentially distributed in duplicated blocks as segmental duplication, while only one pair of gene in *B. juncea* appeared to have undergone tandem duplication (*BjuBCCP8* and *BjuBCCP9*) (Fig. 3).

While identifying the syntenic paralogs on the sub-genomes of *Brassica* as well as their orthologs in *A. thaliana*, we observed that the *BraBCCP1*& *5*; *BolBCCP1* & *6, BniBCCP1* & *5*, and *BjuBCCP1*, *5*, *7* & *11* are orthologs of *Arabidopsis BCCP*-like protein (*BCCPL*-*2*) (Supplementary File 3). In *A. thaliana*, three novel proteins sharing high sequence similarity with the BCCP subunit could be identified; however, unlike the BCCP proteins, they were found not to be biotinylated or lipoylated through immunological assays and their biochemical as well as physiological roles remain to be established. Ding and co-workers [66] found that the plants carrying mutations in each of these *BCCPL* genes grow normally suggesting that these proteins may regulate the expression of other *BCCP* genes.

Clubroot disease is one of the most important diseases of cruciferous plants causing about 10–15% yield loss worldwide [67]. Differential expression of the genes involved in fatty acid and lipid biosynthesis has been reported in roots of *A. thaliana* at both early (4 dpi) and later stages (17 and 20 dpi) of infection by *P. brassicae* [68, 69]. The enhanced expression, probably, due to the accumulation of lipid droplets in plasmodia in the infected root cells of the susceptible plants [69]. We also found an increased expression of majority (7/10) of the *BnaBCCP* members (*BnaBCCP1*, *3*, *4*, *5*, *6*, *9* & *10*) in the roots of susceptible plants, indicating the potential involvement of *BCCP* genes during *P. brassicae* infection (Fig. 5). In contrast, no significant changes in expression level of the *BCCP* genes was observed in roots of the resistant plants. This could be attributed to more accumulation of lipid droplets in susceptible plants over the course of infection [69]. Furthermore, different expression patterns of the *BnaBCCP* genes were observed for root and leaf tissues after clubroot infection. Similar results have been reported by previous studies where genes related to phospholipid synthesis were upregulated in roots of *A. thaliana* while their expression was downregulated in shoots after infection [69]. These results indicate a putative role of BCCP genes in regulating plant’s response to clubroot infection.

*Sclerotinia sclerotiorum* infection is known to induce the oxidative burst of host plant causing the reactive oxygen species (ROS) to attack proteins, lipids, and carbohydrates in the cell, resulting in lipid peroxidation and protein oxidation [70]. In this study, we found four members of the *BCCP* gene whose expression stayed at a reduced levels upon exposure to this pathogen for up to 48 h. Manipulation of these genes through molecular biology approaches, such as genome editing, may improve the resistance to this pathogen. However, functional characterization of these genes, for example, through transformation of *A. thaliana*, will be needed to validate their role in pathogenesis.

Cold is one of the major abiotic stresses, which significantly reduces yield and affects almost every aspect of the physiology and biochemistry of plants by inducing several alterations in cellular components, including changes in the relative amount of unsaturated fatty acids, composition of glycerolipids [71], composition of proteins and carbohydrates, and activation of ion channels [72]. Salt and drought stress are also known to damage the integrity of cell membranes because of increased production of reactive oxygen species [73, 74]. Previously, expression of 16 *BCCP* genes in three *Gossypium* species was reported to show different expression patterns after cold and salt stress [10]. We also found different expression patterns for the same *BCCP* genes following different abiotic stresses. Eight *BnaBCCP* genes showed increased expression at least at one time point after cold and drought stresses indicating that these genes might be co-expressing after these stresses, while expression of only four genes was upregulated after salinity stress. Of the 10 genes, expression of *BnaBCCP6* and *BnaBCCP8* was significantly higher than the controls at the greatest duration of cold, PEG and salt treatments, implying that they might play a fundamental role in response to these stresses.

## Conclusions

In summary, our study provides a comprehensive analysis of the BCCP gene family in the five *Brassica* species, including gene identification, sequence features, physical location, evolutionary relationships, and expression patterns in *B. napus*. This provides valuable information for further elucidation of the evolution and expansion of BCCP gene family. The information obtained from this study provides new insights into potential roles of *B. napus* BCCPs in plant responses to stress and gives valuable gene resources for improving *P. brassicae* and abiotic stress resistance in *B. napus*.

## Methods

### Identification of the *BCCP* family members in *Brassica*

The genome and protein databases of *B. napus* (Brana_Dar_V5), *B. rapa* (Brara_Chiifu_V1.5), *B. oleracea* (Braol_JZS_V1.1), *B. juncea* (Braju_tum_V1.5), *B. nigra* (Brani_San_V1.1) and *B. carinata* (Braca_zd1_V1.0), available at the *Brassica* database (http://Brassicadb.cn/#/) were used in this study. The published *A. thaliana* BCCP amino acid and nucleotide sequences were obtained from the TAIR database (https://www.arabidopsis.org/). To identify the *BCCP* genes, BLASTP and BLASTN searches were performed using the corresponding amino acid or nucleotide sequences from *A. thaliana*. The retrieved non-redundant sequences were submitted to SMART database [75] (http://smart.embl-heidelberg.de/) with chosen option of Pfam [76] domains to confirm each candidate of the *BCCP* gene family. Furthermore, InterProScan program (https://www.ebi.ac.uk/interpro/search/sequence/) [77] was used to validate the initial results for the presence of biotin lipoyl domain in the candidates. Physiochemical properties including the theoretical molecular weight (MW) and isoelectric point (pI) were determined using ExPASy Protparam tool (https://web.expasy.org/protparam/). Subcellular localization of the BCCPs was predicted using the WoLF PSORT (https://wolfpsort.hgc.jp/) [78] and mGOASVM (plant V2) webserver (http://bioinfo.eie.polyu.edu.hk/mGoaSvmServer2/mGOASVM_Plant/) [79] using default parameters.

### Gene structure and conserved motif analysis

The intron/exon organization of the identified *BCCP* genes was determined by comparing their full-length sequences and coding sequences (CDS) using Gene Structure Display Server (GSDS) (GSDS 2.0; http://gsds.gao-lab.org/) [80]. Conserved motifs of the BCCP proteins were identified using online Multiple expectation Maximization for Motif Elucidation (MEME) program (v 5.4.1, https://meme-suite.org/meme//tools/meme) [81]. The MEME parameters were as follows: any number of repetitions, maximum number of motifs 4, and optimum motif width from 6 to 80 amino acid residues.

### Phylogenetic analysis of the BCCP proteins

Multiple protein sequence alignment of the BCCP proteins was performed using Clustal Omega with default parameters. Subsequently, the aligned sequences were used for phylogenetic analysis using MEGA version 7.0 [82]. An unrooted phylogenetic tree was developed using Neighbor joining (NJ) method with pairwise deletion option, Poisson correction method, uniform rates and 1000 bootstrap values.

### Analysis of chromosomal location of the *BCCP* genes and gene duplication

The chromosomal location of each of the *BCCP* gene in *B. napus*, *B. rapa*, *B. oleracea*, *B. juncea* and *B. nigra* was determined based on available genomic information in the *Brassica* database (BRAD), and their distribution on the chromosomes was visualized using Mapchart version 2.2 [83]. Gene duplication events were defined when following conditions were fulfilled: (i) similarity of the aligned region is greater than 80%, and (ii) sequence coverage is more than 80% of the aligned sequence [84]. The segmental duplication events were confirmed when the paralogs were located on duplicated chromosomal blocks on different chromosomes [84].

### Ka/Ks calculations and synteny analysis

The synonymous (Ks) and non-synonymous (Ka) substitution rates of the *BCCP* genes were calculated using MEGA 7.0 software based on coding sequence alignment following the Nei and Gojobori model implemented in MEGA version 7.0 [82]. The divergence time was calculated using the formula T = Ks/2R, where T refers to divergence time, Ks refers to the synonymous substitutions per site, R refers to divergence rate of nuclear genes from plants, where R-value was considered as 1.5 × 10^–8^ synonymous substitutions per site per year in case of dicotyledonous plants [85]. Furthermore, syntenic relationships of the *BCCP*s of *A. thaliana* with *B. napus*, *B. rapa*, *B. oleracea*, *B. juncea* and *B. nigra* were investigated by searching “syntenic gene” function in the *Brassica* database (BRAD) [41].

### Promoter analysis and miRNA prediction

The promoter sequences in the 2 kb upstream regions of the coding sequences for five *Brassica* species were obtained from BRAD, and were analyzed for presence of cis-regulatory elements using the Plant CARE website (http://bioinformatics.psb.ugent.be/webtools/plantcare/html/) [86]. The coding sequences of all *Brassica BCCP*s were submitted to the psRNA-Target server (https://www.zhaolab.org/psRNATarget/) [87] with default parameters to predict miRNAs with a target site on the *BCCP*s.

### Plant materials

Two sets of *B. napus* lines derived from crosses involving canola lines carrying clubroot resistance of the rutabaga (*B. napus* var. *napobrassica*) cv. Polycross and clubroot susceptible *B. napus* canola lines were used to investigate the expression level of the *BnaBCCP* genes after inoculation with the pathogen *Plasmodiophora brassicae* causing clubroot disease. These two sets of lines exhibited resistance or susceptibility to this disease, and each set included 12 lines; all were developed by our research program at the University of Alberta (U of A) from the following cross: [(Polycross × Hi-Q) × A03-74NA] × A03-73NA. Seeds of the parents, the cv. Polycross was obtained from Dr. Dean Spaner, Department of Agricultural, Food and Nutritional Science, U of A, and the cv. Hi-Q and the line A03-74NA and A03-73NA were developed by our program. The details of the development of these 24 lines can be found in Wang et al. [88]. For studies on expression analysis of the *BCCP* genes due to infection by *S. sclerotium*, and exposure to cold, salinity, PEG and hormonal treatments, a spring *B. napus* doubled haploid line “DH12075” was used; this line was expected to be 100% homozygous. Seeds of this line was obtained from Agriculture and Agri-Food Canada, Saskatoon Research Centre, Saskatchewan, Canada.

### Biotic stresses

#### *P. brassicae *infection

The clubroot resistant and susceptible lines were grown in a greenhouse at 22/15 ℃ (day/night) temperature and 16 h photoperiod (8 h dark). For inoculation, resting spore suspensions (inoculum) of *P. brassicae* pathotype 3A from the preserved galls were prepared following the protocol described by Strelkov et al*.* [89] and the concentration of the suspension was adjusted to 1 × 10^7^ to 1 × 10^8^ resting spores/mL. Seedlings of each of the resistant and susceptible lines were grown in 3 cm × 3 cm × 5 cm (L × W × D) cells filled with Sunshine® Professional Growing Mix (Sungrow Horticulture, Agawam, MA, USA 01,001), and 10-days old seedlings were inoculated with 1 mL of spore suspension. In case of control, seedlings of each of these lines were grown under same condition and were inoculated with water. Bulk root and leaf samples of the resistant and susceptible lines from the control and infected treatments were harvested at 7, 14 and 21 days after inoculation (dai), snap frozen in liquid nitrogen and stored at -80 ℃ until used. Thus, the total number of bulks was 2 type lines × 2 treatments × 3 time points = 12, where each bulk included 48 plants. The experiment was repeated three times which constituted three replications.

#### *S. sclerotium *infection

For this, DH12075 plants were initially grown in a greenhouse under the condition mentioned above, and 20 days old plants were transferred to a humidity chamber for 24 h prior to inoculation. For inoculation, *S. sclerotiorum* cultures were grown on potato dextrose agar medium (PDA; Becton Dickinson, Columbia, MD) and incubated at room temperature (21 ± 2 °C) for 3 days. The true leaves of the plants were inoculated with mycelial plugs (5 mm), while for control, the leaves were inoculated with sterile PDA plugs without fungal cultures. The details of the inoculation technique can be found in Joshi et al*.* [27]. The leaf samples of the control and inoculated plants were harvested at 12, 24 and 48 h after inoculation (hai) and frozen immediately in liquid nitrogen. Three independent biological replicates were carried out, and each replicate included 20 plants at each time point and two leaves per plant were used.

### Abiotic stresses

#### Cold stress (CS)

For this, 3-week-old DH12075 plants, grown in a greenhouse at 22/18 °C (day/night) temperature and 16/8 h photoperiod, were placed in a growth chamber set at 4 °C constant temperature and constant light with an intensity of 45–55 μmol m^−2^ s^−1^ at plant level. Fully expanded second and third leaves were harvested after 1, 2 and 7 days of cold treatment, frozen in liquid nitrogen, and stored at − 80 °C until use. At the same time, the second and third leaves of the unstressed plants grown in the above-mentioned greenhouse were harvested as control. For each time point and each of the two treatments (control and cold stress) bulk sample of five plants were used, and the experiment was repeated three times which constituted three replicates [28].

#### Salinity and drought stress

For this, 3-week-old DH12075 plants grown in a greenhouse at 22/18 °C (day/night) temperature and 16/8 h photoperiod were used. For inducing drought stress, each plant was sprayed with 20 ml of 20% (w/v) polyethylene glycol (PEG 8000) using a spray bottle, and the control plants were sprayed with distilled water. For salinity stress, the plants in nine by eight cells trays (cell size: 4 × 4 × 5 cm, length × width × height) were filled with 350 ml of 125 mM NaCl solution, while the control plant trays were filled with normal water. The treated plants for both stresses were kept in the same greenhouse, and the PEG treated plants were watered normally after 24 h while the salt treated plants were not watered after the stress. The control and treated leaf samples were harvested at 6, 24 and 48 h after the treatments and were flash frozen in liquid nitrogen and stored at -80 °C. For each time point 2^nd^ and 3^rd^ leaf of 10 plants were pooled and flash frozen, and the experiment was repeated three times.

#### Hormonal treatments

For hormonal treatments, 3-week-old DH12075 plants grown in the above-mentioned greenhouse were used, and the plants were sprayed with 5 ml of ABA, SA and BAP solutions following Yang et al*.* [90]. Briefly, ABA was first dissolved in absolute ethanol to prepare a 20 mM stock solution and then diluted with 0.1% (v/v) ethanol to the final 50 μM solution used to spray the plants. SA was dissolved in distilled water to prepare a 100 mM stock solution with the adjustment of pH to 6.5 using 1 M KOH before dilution in distilled water to the 1 mM working solution. BAP was dissolved in 1 M NaOH to prepare a 1 mM stock solution after which it was diluted with distilled water to the 20 μM working solution. Control treatments included 0.1% (v/v) ethanol for ABA and distilled water adjusted to pH 6.5 with 1 M KOH or 1 M NaOH for SA or BAP treatments. Control and treated leaf samples were harvested at 6, 24 and 48 h after spray, flash frozen in liquid nitrogen and stored at -80 °C. For each time point 2^nd^ and 3^rd^ leaf of 10 plants were pooled and flash frozen. The experiment was repeated three times.

### RNA isolation and Quantitative Reverse Transcription-Polymerase Chain Reaction (qRT-PCR)

Total RNA was isolated from the frozen control and treated samples using TriZol reagent and treated with DNAse 1 (Promega) to remove contaminating DNA according to manufacturer’s instructions. The quality and concentration of the RNA was determined using a NanoDrop-1000 spectrophotometer. RNA samples with 260/280 nm ratio of 1.8–2.0 and 260/230 nm ratios ≥ 2.0 were used for further analysis. Approximately 2 µg total RNA was used for first strand cDNAs synthesis with the RevertAid First Strand cDNA Synthesis Kit (ThermoFisher Scientific) following the manufacturer’s instructions. Gene-specific primer pairs were designed using Primer Express v3.0.1 (Life Technologies, ON, Canada) based on CDSs of the *BCCP* genes. The sequences of the primer pairs are listed in Supplementary File 1. qRT-PCR was performed on a StepOne Plus real time PCR system (Life Technologies, Burlington, ON) using FASTSYBR Green mix from ThermoFisher Scientific. Three biological replicates for each sample and two technical replicates of each biological replicate were analyzed. PCR amplification conditions used were: 95 ℃ for 2 m followed by 45 cycles of denaturation at 95 ℃ for 10 s, annealing, and elongation at 60 ℃ for 35 s. The constitutively expressed housekeeping gene *Ubiquitin-Conjugating Enzyme 9* (*UBC9*) from *B. napus* was used as endogenous control. The changes in the relative expression levels of the *BCCP* transcripts were calculated using the 2^− ΔΔCt^ method [91].

### Statistical analysis

For all treatments, control sample of each time point was used as a calibrator with relative expression equal to 1. One- and two-way ANOVA were employed for treatments using DH12075 and two bulks, respectively. The significance of differences with *p* < 0.05 among the relative expression levels of the genes was analyzed by Tukey HSD test.

## Supplementary Information

**Additional file 1.**

**Additional file 2.**

## Data Availability

All data generated or analysed during this study are included in this published article and its supplementary information files.

## Abbreviations

BCCP: Biotin carboxyl carrier protein
ACCase: Acetyl CoA-carboxylase
CDS: Coding Sequence
Ks: Synonymous substitution rate
Ka: Non- synonymous substitution rate
MW: Molecular weight
pI: Isoelectric point
NaCl: Sodium chloride
PEG: Polyethylene glycol
ABA: Abscisic acid
SA: Salicylic acid
BAP: Benzyl aminopurine
