# Supplementary material for: Genome-wide identification of biotin carboxyl carrier subunits of acetyl-CoA carboxylase in Brassica and their role in stress tolerance in oilseed Brassica napus
Source: BMC Genomics. 2022 Oct 17;23:707. doi: 10.1186/s12864-022-08920-y (PMC9578262; doi:10.1186/s12864-022-08920-y)
Supplement: Supplementary file 1 — Additional file 1. [file 12864_2022_8920_MOESM1_ESM.docx]

Supplementary Figure 1: Phylogenetic relationships of *BCCP* genes in each of *B. rapa*, *B. oleracea*, *B. nigra,* *B. napus* and *B. juncea.* Ten unrooted phylogenetic trees (a – j) were generated using Neighbor-Joining (NJ) method.





a





b





c





d





e





f





g





h





i





j
